# Callous–Unemotional Traits and Emotion Perception Accuracy and Bias in Youths

**DOI:** 10.3390/children11040419

**Published:** 2024-04-01

**Authors:** Enrica Ciucci, Andrea Baroncelli, Carolina Facci, Stefania Righi, Paul J. Frick

**Affiliations:** 1 Department of Education, Languages, Interculture, Literatures and Psychology, University of Florence, Via di San Salvi 12, Complesso di San Salvi Padiglione 26, 50135 Florence, Italy; enrica.ciucci@unifi.it; 2 Department of Philosophy, Social Sciences and Education, University of Perugia, Piazza Giuseppe Ermini 1, 06123 Perugia, Italy; andrea.baroncelli@unipg.it; 3 Department of Neuroscience, Psychology, Drug Research and Child’s Health, University of Florence, Via di San Salvi 12, Complesso di San Salvi Padiglione 26, 50135 Florence, Italy; stefania.righi@unifi.it; 4 Department of Psychology, Louisiana State University, 208 Audubon Hall, Baton Rouge, LA 70803, USA; pfrick@lsu.edu

**Keywords:** callous–unemotional traits, conduct problems, emotion recognition accuracy, emotion recognition bias, middle school

## Abstract

This study investigated the associations among conduct problems, callous–unemotional (CU) traits, and indices of emotion recognition accuracy and emotion recognition bias obtained from human faces. Impairments in emotion recognition were considered within broader, impaired emotional and social functioning. The sample consisted of 293 middle-school students (51.19% girls; M age = 12.97 years, SD = 0.88 years). In general, CU traits were associated with less accuracy in recognizing emotions, especially fearful and angry faces, and such deficits in emotional recognition were not associated with conduct problems independent of CU traits. These results support the importance of studying potential deficits in the recognition of emotions other than fear. Furthermore, our results support the importance of considering the role of CU traits when studying emotional correlates of conduct problems. For children scoring high on CU traits, the emotion recognition accuracy of anger was low irrespective of the level of conduct problems, whereas in children scoring low on CU traits, less accuracy in recognizing emotions was related to increases in conduct problems. Finally, our results support the need for research to not only focus on accuracy of emotional recognition but also test whether there are specific biases leading to these inaccuracies. Specifically, CU traits were associated not only with lower accuracy in recognizing fearful faces but also with a tendency to interpret fearful faces as angry. This suggests that the emotional deficit associated with CU traits is not just a deficit in empathic concern toward others distress but also includes a tendency to overinterpret emotions as potential threats to oneself.

## 1. Introduction

Callous–unemotional (CU) traits represent affective components of the conscience and the affective features of psychopathy [1]. They designate a particularly aggressive subgroup of youths with severe early-onset conduct problems and are characterized by reduced empathy and guilt for misdeeds, lack of concern about performance in important activities, and restricted affect [1,2]. CU traits have been included in the fifth edition of the DSM as a specifier for serious conduct problems (i.e., conduct problems with limited prosocial emotions; American Psychiatric Association [3]). Specifically, youths with strong CU traits show distinct genetic, emotional, cognitive, and social characteristics, as confirmed by research on both clinical and community samples of youths [2,4,5,6]. The aim of this study was to contribute to the understanding of the role of CU traits in the processing of others’ emotions in terms of both recognition accuracy and recognition bias within a community sample of middle school students, considering the transition from childhood to adolescence a time of rapid biological, psychological, and relational changes that may impact the development of personality traits [7]. Specifically, we explored the unique and shared role of conduct problems and CU traits in (a) emotion recognition accuracy for both a set of emotion faces (i.e., happiness, sadness, anger, fear, and neutral emotion) and with specific reference to anger and fear faces, (b) emotion recognition bias concerning anger and fear faces, and (c) the specific anger-to-fear and fear-to-anger biases.

### 1.1. Emotion Recognition and CU Traits

Emotion recognition is a key cognitive ability that supports human social information processing: the ability to recognize others’ emotions is important for the understanding of others’ intentions, the prediction of their behavior, and the initiation of the empathic process, while impairments in emotion recognition alter accuracy in making social interpretations and lead to maladaptive outcomes such as aggression toward others [8,9,10,11,12,13]. There is also consistent evidence suggesting that CU traits are associated with impairments in recognizing and responding to emotional cues from others [1]; this was robustly documented across different emotional cues (e.g., emotional picture, vocal tones, body posture, and words), mode of presentation, and key features in emotional faces (e.g., eye gaze direction) [14,15,16,17,18,19,20,21,22,23]. One commonly accepted explanation for this reduced emotion recognition relies on the differential patterns of attention to facial cues. Compared to typically developing individuals, those with strong CU traits have difficulty in directing attention to emotionally salient aspects of faces and, particularly, look less to the eyes [18,19,24]. This mechanism is part of a broader impaired emotional functioning, also involving an attenuated functioning of the anterior insula and amygdala, related to reduced autonomic reactivity to distress cues in others [25].

While some impairment in recognition is often found in research as being related to CU traits, the extent of the impairments is unclear. For instance, while some studies found that impairment was evident to some extent across many different emotions [5,14,22,26,27,28], other studies mainly focused on deficits in the processing of negative emotional stimuli [19], and, in particular, facial depictions of distress, such as sadness [23,24,29] and fear [15,21,29,30]. Moreover, not all studies have confirmed these impairments; for instance, some studies have found a positive association between CU traits and fear recognition [24,31,32,33]. In addition, Hartmann and Schwenk [34] found that CU traits were associated with the slower recognition of angry, sad, and fearful faces but not with higher error rates, suggesting that emotion recognition deficits could depend on deficits in processing speed.

One possible approach to better clarifying the association between CU traits and emotion recognition ability is to consider their relationship with conduct problems more generally. In their recent meta-analysis involving 23 fMRI studies, Berluti and colleagues [35] investigated the responses to aversive stimuli (fearful and angry facial expressions and empathic pain stimuli) among youths with conduct problems, finding that a reduced activation in the right amygdala to negative images and fearful facial expressions was present across youths with conduct problems, rather than being limited to youths with stronger CU traits. Given that CU traits and externalizing behavior are highly correlated [36] but that CU traits may designate a subgroup of youths with conduct problems who differ on their emotional correlates [2], it is important for researchers to consider both the shared and unique correlates related to CU traits and conduct problems [2]. For example, Leno and colleagues [26] found that CU traits were associated with reduced emotion recognition accuracy across all emotion in an uncued condition (i.e., no fixation cross to the eyes presented), but, when adjusting for conduct problems, this association was no longer present. However, CU traits were associated with better recognition of fear when cued to the eyes, even when adjusting for conduct problems. These results support the importance of accounting for the shared variance between conduct problems and CU traits. Furthermore, it would also be important to test the interactive role of conduct problems and CU traits in the associations with emotion recognition ability, since a growing field of studies suggests that children or adolescents with more conduct problems but normative in levels of CU traits show no deficits in their emotion recognition processing and may in fact present an enhanced emotional responsiveness to distress cues in others (i.e., emotional hyperreactivity), while those with both conduct problems and strong CU traits present deficits in emotional reactivity to others’ distress (i.e., emotional hyporeactivity) [1,2,6].

Another potentially important way to advance research into the emotion recognition impairments related to CU traits is to not just simply document levels of accuracy but to consider possible reasons for this inaccuracy in terms of specific emotional biases. Emotion recognition accuracy corresponds to accurately labeling a discrete emotion when it is expressed (e.g., accurately labeling anger when a face expresses anger), while emotion recognition bias corresponds to perceiving a particular discrete emotion when a different emotion is expressed (e.g., labeling a fearful face as angry) [37]. According to Barth and Bastiani [38], perception bias could play a more significant role than recognition accuracy in predicting social behavior; when individuals are prone to over-reading a specific emotion, they are more likely to distorted processing of social information and adopt maladaptive behavioral decisions. For instance, socially rejected and aggressive children are prone to show a biased attribution of specific emotions (i.e., anger in an ambiguous situation or happiness to provocateurs in a provocative situation) that lead them to over-detect threats in the environment and to choose attack strategies [8,39,40,41]. While previous research has found that youths with concomitant conduct problems and CU traits are less affected by attentional biases toward emotional stimuli in tasks concerning dot-probe or lexical decision [42,43], as far as we know, the relationship between CU traits and emotion recognition bias in a face recognition task has never been explored. 

### 1.2. This Study

Based on this past work, in the present study, we tested the unique and interactive role of conduct problems and CU traits in the emotion recognition of a set of faces expressing different emotions (i.e., happiness, sadness, anger, fear, and neutral emotion). We expected that CU traits, but not conduct problems, would be negatively associated with emotion recognition accuracy. We also tested emotion recognition accuracy specific to fearful and angry emotions. While most past research has focused on facial depictions of distress, there is research highlighting that those with CU traits show reduced attention to threats in the environment (i.e., low arousal to threat) [44]. Since the sight of angry faces can activate individuals in a self-defensive way (i.e., flee from those who show any anger that could potentially lead to aggression), we hypothesized that CU traits would be negatively associated with the accurate recognition of angry stimuli as well. 

As for emotion recognition bias, we once again focused on anger and fear emotions. Since no previous studies concerning face recognition task exist, we explored the unique and interactive role of conduct problems and CU traits, and we made our predictions based on studies that focused on other emotional tasks [42,43,44], hypothesizing that those with conduct problems would be associated with more recognition bias especially at low levels of CU traits, since this group has shown a hostile attribution bias in past research. Furthermore, we explored two specific emotion recognition biases concerning the over-reading of anger in front of faces expressing fear and the over-reading of fear in front of faces expressing anger. 

In the present study, we also tested the potential moderating role of age and sex. As for age, Frick and Kemp [45] noted that studies have generally found negative associations between CU traits and emotion recognition accuracy in young children [15,33], while there have been more inconsistent results in samples of older children and adolescents [23,31,32,33]. According to Frick and Kemp [46], deficits in emotional reactivity may hinder the development of emotional recognition skills in young children with elevated CU traits, but, over time, the ability to use emotions for social gain may motivate them to acquire emotional recognition abilities. Thus, in the present study, we tested the hypothesis that the above-predicted role of CU traits in emotion recognition accuracy and emotion recognition bias would be stronger at younger ages. As for sex, the existing literature has suggested that girls have an advantage in facial expression recognition due to greater attention to the eyes [46]. Furthermore, Winters and Sakai [28] reported that CU traits were more strongly associated with problems in emotion recognition in boys. Thus, we explored the possible moderating role of sex and predicted that association between CU traits with emotion recognition accuracy and emotion recognition bias would be stronger for boys. 

Finally, in the present study, we also considered the potential impact of the direction of eye gaze in the facial expression we used in the emotion recognition task. Direction of eye gaze has been shown to influence the recognition of emotional facial expression and the subsequent cognitive processing of the emotion [47]. According to the shared signal hypothesis (SSH) [48], when there is congruency between gaze direction and the underlying behavioral intent communicated by the expression of a specific emotion, it enhances the perception of that emotion. A gaze usually indicates interest in terms of approach–avoidance, since people often look at things they like and avoid things they do not [49,50]. Angry people often stare into the eyes of the person with whom they are fighting or quarrelling; happy people often look to the eyes of the person with whom they want to approach; people who fear others often look away from their eyes. So, a direct eye gaze enhances the perceived intensity of approach emotions (such as anger and happiness), whereas an averted gaze enhances the perceived intensity of avoidance emotions (such as fear and sadness) [48,51,52]. These effects were found for static faces in adult samples [48,51,52], and they have been replicated in child samples [53]. Bedford and colleagues [14] tested emotion recognition difficulties in relation to CU traits for both static and dynamic stimuli and whether emotion recognition performance was moderated by eye-gaze direction. For static facial expressions, they found that stronger CU traits were significantly associated with reduced emotion recognition for angry and happy faces. As far as we know, no study has investigated whether the association between CU traits and emotion recognition performance is moderated by face direction. Thus, in the present study, we tested this possible influence of face direction in an exploratory manner.

## 2. Materials and Methods

### 2.1. Participants and Procedure

The present research was conducted in a convenience sample of middle school students in central Italy. Specifically, two public schools were contacted to take part in research on emotional development and socioemotional adjustment among middle school students, taking into account that this sample was a group of preadolescents in a phase of change that can have an effect on their personality development [7]. Internal school boards approved all procedures, and written parental consent was requested for over 350 students; no economic incentives were given. Data collection was conducted during school hours by trained assistants: students with parental consent were invited to individually complete a paper questionnaire during a group session. Due to the need to provide supervision of children of middle school age, group sessions were kept modest in size, and a researcher was present to monitor behavior and answer questions. Also, due to the age of the sample, the emotion recognition task was administered to one student at a time in a room that the school had specifically made available for this purpose. Each participant was provided a code number to control for confidentiality and privacy. Prior to data coding, the following exclusion criteria were applied: unavailability declared by the student to participate in the research; inability to understand written text, even if supported (i.e., presence of an intellectual disability or unfamiliarity with the Italian language); partial completion of measures of the tools; multiple and repeated school failures that placed a student outside of the typical age range (i.e., middle school in Italy is attended by students aged between 11 and 14 years). Consequently, the final sample consisted of 293 students (51.19% girls; M age = 12.97, SD = 0.88; 90.44% of them were of Italian cultural background; the distribution across the three school grades was 43 grade 6 (14.68%), 128 grade 7 (43.69%), and 122 grade 8 (41.64%) students. This sample was diverse regarding parental educational level but representative of families in the school area: 168 fathers (57.34%) and 196 mothers (66.89%) reported a high school or a university degree, in line with the data indicating that 62.70% of Italians between 25 and 64 years hold at least a high school degree [54].

### 2.2. Measures

#### 2.2.1. Inventory of Callous–Unemotional Traits (ICU)

CU traits were assessed using the Inventory of Callous-Unemotional Traits (ICU) [36], with the Italian version validation by Ciucci and colleagues [4]. This is one of the tools most widely used to assess these traits worldwide [55]. It is a 24-item self-report questionnaire (e.g., “The feelings of others are unimportant to me”; “I try not to hurt others’ feelings” (reversed); “I hide my feelings from others”), to be completed using a 4-point Likert-type scale, from “not at all true” (0) to “definitely true” (3). A total score of CU traits was obtained by the mean of all items (excluding items 2 and 10, as per Ciucci and colleagues [4]). The Cronbach’s alpha in the present sample was 0.82.

#### 2.2.2. Conduct Problems

Conduct problems were investigated using a subscale of the Strengths and Difficulties Questionnaire (SDQ) [56]. The subscale is made up by 5 items (e.g., “I fight a lot. I can make other people do what I want”; “I get very angry and often lose my temper). Participants rated each item using a 3-point Likert-type scale (from 0 = “not true” to 2 = “certainly true”), and a total score was obtained as the mean of all items. The Cronbach’s alpha in the present sample was 0.45. Despite the low alpha value, we adopted this measure of conduct problems because it is a widely-used tool in worldwide research with significant support for the validity of its subscales [57,58], and there is substantial evidence in the extant literature that the SDQ, in general, and the conduct problems subscale, in particular, show significant associations with important external criteria [58,59]; specifically, Goodman and colleagues [60] reported high levels of agreement between SDQ scores and independent clinical diagnoses of several psychiatric categories (including conduct disorders).

#### 2.2.3. Recognition Task 

A total of 24 face identities (12 female) were taken from the Dartmouth Database of Children’s Faces [60], which are face stimuli commonly used to estimate the prevalence of face and emotion recognition deficits in children [61,62]. The mean estimated age of the selected identities was 9.47 years (SD = 2.49). In the Dartmouth Database of Children’s Faces, models were photographed on a black background and were wearing black bibs and black hats to cover hair and ears. For each identity, the photographs (totaling 120 faces) comprised 5 emotions: neutral, angry, fearful, sad, and happy expressions. Emotional faces were frontal facing with frontal gaze (60 faces) or 30° left-oriented (60 faces). Faces were embedded in a rectangular frame that measured 8.5 cm by 5.5 cm, and they were centrally presented on the screen in a random way on a black background for 500 ms. The fixation cross between the faces lasted 600 ms. Subjects were requested to classify facial expression by pressing 1 of 5 buttons on the keyboard. The emotion recognition task was divided in 6 blocks of 20 faces for a total of 120 faces presented. Before the experimental section, subjects were trained with three faces (3 different identities) taken from Dartmouth Database of Children’s Faces Models (1 oriented, 2 frontal gaze) with angry, neutral, and happy expressions, respectively.

Emotion recognition accuracy (all emotions, anger, and fear; frontal and oriented) and emotion recognition bias (anger and fear) scores were calculated according to Fine and colleagues [37]. Specifically, for each emotion recognition accuracy score, the number of correct responses was first calculated and then squared; this score was then divided by the number of faces depicting each target emotion (i.e., all emotions, anger, and fear; frontal and oriented) multiplied by the number of times that a participant identified the target emotion across all stimuli. The emotion recognition accuracy scores thus reflect participants’ accuracy in identifying each target set of stimuli, corrected for chance. Moreover, to calculate emotion recognition bias for angry and fearful faces (both frontal and oriented), the number of times that a participant answered a targeted emotion (e.g., anger) for nonanger faces was divided by the participant’s total number of incorrect responses made for nonanger faces. Therefore, the emotion recognition bias scores represent the percentage of times a participant chose a specific emotion as an incorrect answer out of all incorrect answers to all other faces expressing the other emotions. Finally, specific “anger to fear” and “fear to anger” emotion recognition biases (both frontal and oriented) were calculated as count variables, i.e., by summing the number of times a participant made that specific error. 

### 2.3. Data Analyses

Descriptive statistics and zero-order correlations (i.e., Pearson’s *r*) for all study variables were calculated. To test the unique effects between CU traits and conduct problems, as well as potential interactions with sex and age, a series of multiple regression analyses were conducted for all, emotion recognition accuracy, and emotion recognition bias variables. Specifically, the emotional indices were the dependent variables, whereas sex, age, conduct problems, and CU traits were the independent variables in Step 1. The two-way interaction term conduct problems × CU traits was added in Step 2, and the other two-way interaction terms (e.g., conduct problems × sex and CU traits × sex) were added in Step 3. Finally, the three-way interaction term (conduct problems × CU traits × sex was tested in Step 4. Steps 3 to 4 were reperformed replacing sex with age. For any significant interaction, the form of the interaction and significance of simple effects were explored using the post hoc probing procedures suggested by Holmbeck [63]. Specifically, the regression equation derived from the full sample was used to estimate predicted values for the dependent variable at one SD below and one SD above the mean. Prior to computing interaction terms, scores were centered by subtracting the sample means. Given the highly skewed count nature of the specific bias “anger to fear” and “fear to anger”, we adopted negative binomial regressions for analyses using these dependent variables. We checked for multicollinearity by calculating the variance inflation factor (VIF) of each independent variable, which represents the extent to which correlations with other independent variables affect its variance. Considering the threshold range of VIF > 3, our results were not affected by significant multicollinearity because no VIF was >2.55.

## 3. Results

Descriptive statistics and zero-order correlations are reported in Table 1. As noted in Table 1, age was not related to the indices of emotional accuracy, but boys were less accurate in interpreting emotions than girls, which is consistent with the findings of past research [64]. Correlations involving the recognition task were similar in magnitude and in direction for frontal and oriented stimuli. As result, these were combined for the test of the main study hypotheses. Consistent with our hypotheses, CU traits were negatively correlated with emotional recognition accuracy for all emotions (*r* = −0.12; *p* < 0.05), as well as for recognizing fear (*r* = −0.15, *p* < 0.01) and anger (*r* = −0.20, *p* < 0.001). Conduct problems were also negatively related to emotional recognition accuracy for all emotions (*r* = −0.12, *p* < 0.05) but not specifically to accuracy in identifying angry and fearful faces. Finally, CU traits (*r* = 0.24, *p* < 0.001) and conduct problems (*r* = 0.14, *p* < 0.05) were significantly correlated with an anger bias to fearful faces, whereas only CU traits were associated with a fear bias for interpreting angry faces (*r* = 0.14, *p* < 0.05).

**Figure 1:**
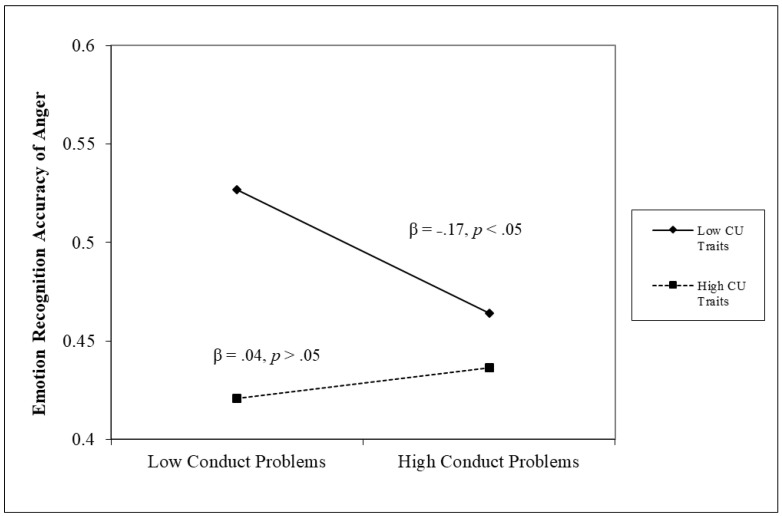
Conduct problems × cu traits interaction term in anger recognition accuracy.

The results of linear regression analyses testing unique and interactive effects are reported in Table 2. For the analyses of emotion recognition accuracy, there only emerged the unique negative role of CU traits for all emotions (β = −0.13, *p* < 0.05) and anger (β = −0.15, *p* < 0.05). Importantly, there was a significant two-way interaction between conduct problems and CU traits in analyses with anger recognition (β = 0.13, *p* < 0.05). This interaction is plotted in Figure 1 and indicates that, for those with strong CU traits, the emotion recognition accuracy of anger was not related to conduct problems (β = 0.04, *p* > 0.05), whereas for those with weak CU traits, conduct problems were negatively related to emotion recognition accuracy (β = −0.17, *p* < 0.05). 

**Figure 2:**
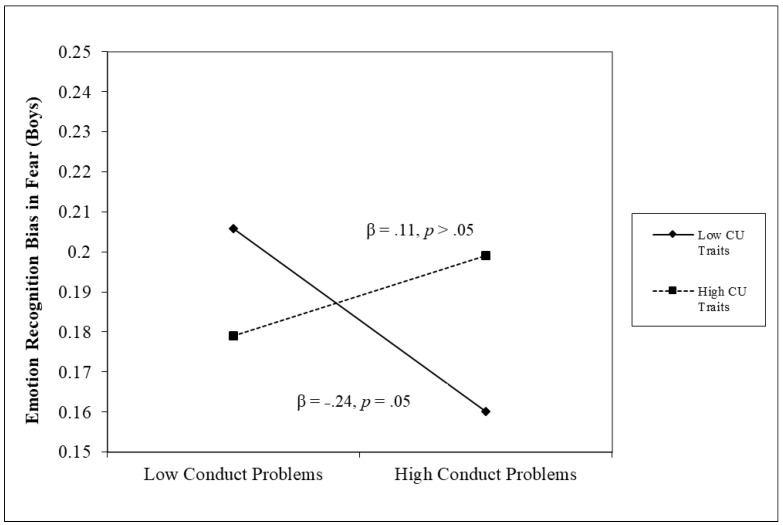
Conduct problems × CU traits interaction term in emotion recognition bias in fear (in boys).

For the analyses of emotion recognition bias for fear, no unique main effects of conduct problems or CU traits emerged. However, there was an unexpected significant three-way interaction for conduct problems × CU traits × sex in emotional bias of fear (β = 0.19, *p* < 0.05). Follow-up analyses indicated that the conduct problems × CU traits interaction was significant in boys (β = 0.21, *p* < 0.05) but not in girls (β = −0.07, *p* > 0.05). The form of this interaction in boys is shown in Figure 2. As shown in this figure, conduct problems were negatively associated with fear bias at low levels of CU traits (β = −0.24, *p* = 0.05) but positively associated at high levels (β = 0.11, *p* > 0.05), although neither slope reached statistical significance.

The results of negative binomial regression analyses testing indices of specific recognition biases (i.e., anger to fear; fear to anger) as the dependent variable are reported in Table 3. Stronger CU traits were associated with a higher propensity to read anger in front of fear (β = 0.70, *p* < 0.05). Furthermore, none of the interactions tested reached statistical significance. 

## 4. Discussion

Impairments in emotion recognition can impair children’s development of empathy by undermining their accuracy in understanding others’ intentions and promoting maladaptive interactions with others; as a result, it has been proposed as a critical explanatory mechanism of the cause of CU traits [8,9,12,13,45]. Considering the potential causal role of emotion recognition and trying to clarify past inconsistencies on the association between CU traits and emotion recognition skills, a growing body of research has suggested the need to consider the co-occurring role played by other variables, such as sex, age, and conduct problems [1,2,6,26,35]. Thus, in the present study, using a community sample of middle school students, we tested the unique and interactive effects of sex, age, conduct problems, and CU traits in their associations with the child’s ability to correctly recognize the emotions in a set of faces expressing different emotions (i.e., happiness, sadness, anger, fear, and neutral emotion), with particular attention to angry and fearful faces. We decided to focus largely on anger and fear based on past research suggesting that CU traits may be specifically related to reduced attention to distress in others [6] and threats in the environment [44]. Importantly, for the first time, we also considered possible explanations of any emotion recognition impairments in terms of emotion recognition bias (i.e., the tendency to systematically perceive a particular discrete emotion when a different emotion is expressed), again with specific reference to the over-reading of anger in front of faces expressing fear and the over-reading of fear in front of faces expressing anger. Finally, another novel aspect of the present study was considering the potential impact of face direction (i.e., frontal vs. oriented) in the facial expression on any deficits associated with CU traits. 

Our results supported several hypotheses. First, we found that both conduct problems and CU traits were negatively related to an index of emotion recognition accuracy across emotions (i.e., happiness, sadness, anger, fear, and neutral emotion); however, regression analyses indicated only the significant unique role of CU traits remained when CU traits and conduct problems were tested together in multiple regression analyses. Specifically, in line with recent studies [5,14,22,26,27], this evidence further supports the notion that children strong in CU traits have difficulty with emotion recognition, and this could be related to their difficulty in directing attention to emotionally salient aspects of faces, such as the eyes [18,20,24]. Furthermore, these results suggest that this emotional deficit seems to be more specific to CU traits and that research on association between conduct problems and emotion recognition accuracy needs to consider the influence of these traits [2]. 

In addition to our testing of the accuracy in identifying emotions more generally, we focused specifically on two emotions (i.e., fear and anger) that could be especially important for understanding the development of CU traits and for understanding how these traits lead to serious conduct problems. As we predicted, we found that CU traits were negatively associated with fear recognition, and this finding is consistent with those of a host of past research indicating a “fear blindness” for youths strong in CU traits [17,24,32,33]. Importantly, conduct problems were not associated with accuracy in recognizing fear, even in zero-order correlations (see Table 1), suggesting that this difficulty recognizing fear may be even more specific to CU traits. However, contrary to our hypotheses, this association was not moderated by sex, as predicted by Winters and Sakai [28], nor was it moderated by age, as predicted by Frick and Kemp [45]. That is, Frick and Kemp [45] proposed that emotional deficits may become less associated with CU traits as child ages and learns that recognizing emotions may have value in social interactions, particularly allowing a child to be more successful in manipulating others. However, the failure to find such an interaction may have been due to the very limited age range of our sample.

An important advance provided by our study was examining not only accuracy in emotional recognition, which has been the focus of many past studies, but also emotional recognition biases, which have not been the focus of much past work in relation to CU traits. While our results did not show a consistent bias to fear (i.e., a tendency to attribute fear to other emotions) related to CU traits, there was a significant interaction between conduct problems and CU traits that was unique to boys. Specifically, only in boys, conduct problems were negatively associated with bias in fear recognition but only at low levels of CU traits. Importantly, this interaction was not predicted a priori and, as a result, needs to be interpreted cautiously. However, these findings would be consistent with the violence inhibition mechanism (VIM) model of aggression [65], suggesting that observing distress in others (such as seeing fear in the eyes of others) promotes empathy and inhibits responses that would harm others. Our results, if replicated, would suggest that this mechanism may only be operating for those with weak CU traits. In our sample, an increase in a tendency to overinterpret fear cues in others led to more conduct problems in those with strong CU traits, which could mean that in this group, fearful cues in others could signal an increased vulnerability and susceptibility to be attacked rather than signaling a need for empathy [23]. Importantly, while the significant interaction does suggest that interpreting fear cues differ in their relationship with conduct problems depending on the level of CU traits in boys, neither of these simple slopes reached significance. 

Another important advance provided by our study is the focus on the emotional recognition of anger, as well as fear. Overall, problems recognizing anger was also uniquely associated with CU traits and not conduct problems. These findings are consistent with those of Munõz [21], who reported that high levels of CU traits were related to more errors in labeling angry faces. These results are also consistent those of with a model proposed by Waller and Wagner [66], in which low threat sensitivity (e.g., ignoring signs of anger toward ones’ own behaviors on the part of parents, teachers, or peers) is considered an important causal factor to CU traits. These findings are important because research on emotional accuracy has not always focused on anger recognition, instead focusing more on the accurate recognition of fearful and sad faces. However, unlike for fear, we found a significant interaction between conduct problems and CU traits when testing anger recognition accuracy. Specifically, for those with strong CU traits, anger recognition accuracy was low overall and unrelated to the level of conduct problems (see Figure 1). In contrast, in those with weak CU traits, greater accuracy in recognizing anger led to fewer conduct problems. Given that this result was not predicted, it again should be interpreted cautiously. However, it illustrates the importance of considering CU traits when studying the emotional correlates of conduct problems, given that deficits associated with CU traits (e.g., problems recognizing anger) may mask deficits that may lead to conduct problems in those with weak CU traits. 

Finally, our findings related to bias toward interpreting anger may help to further clarify the emotional deficits associated with CU traits. That is, not only were CU traits associated with less accuracy in recognizing angry faces, they were also related to a tendency to interpret fearful faces as angry (see Table 3). This association with an anger bias to fearful faces was not modified by age, sex, or conduct problems. This finding was also not predicted a priori and, in fact, was somewhat opposite to our prediction that conduct problems would be associated with a “hostile attribution” bias only at low levels of CU traits. However, this prediction was based on only a few studies using other emotional paradigms and not an emotional recognition task [42,43]. Instead, our results suggest that CU traits are associated with a proneness to interpret fearful faces as angry. This finding could be explained by the fact that fearful faces reflect distress in others and signal the need to for empathetic responses toward others [38]. As noted in our results and in those of others, CU traits are related to lower accuracy in interpreting fear in others, and this deficit could contribute to the low levels of empathy often associated with these traits [45]. However, our results, if replicated, suggest that CU traits are not only related to this difficulty interpreting others distress: those with CU traits also have a tendency to interpret this distress as anger, which is a cue to a threat to self [43]. Such a tendency to overinterpret emotional cues as threats could lead children with strong CU traits to assume an attack-like and dominant-prone attitudes toward others, which further contributes to their tendency to ignore cues of potential harm to others and to act aggressively toward them. 

All of our results need to be evaluated considering several limitations. The sample of Italian school children used in this study was relatively homogenous with respect to ethnic backgrounds, as is typical in Italian schools. Thus, the generalizability of our results to other samples in countries with different cultures, races, and ethnicities may be limited. Moreover, the cross-sectional nature of our data did not allow for the direction of causality to be determined. Longitudinal studies are needed to determine the direction of causality between emotion processing deficits (both accuracy and bias) and CU traits. For example, CU traits may lead a child to engage in harmful behaviors toward others, which often result in angry responses from others. These interactions could lead children with strong CU traits to expect angry responses from others, accounting for their bias toward interpreting fearful faces as angry. Also, the age range of our sample may have influenced our findings. As noted above, while Frick and Kemp [45] proposed potential developmental changes in the associations between CU traits and emotion recognition, our sample was relatively homogeneous in terms of age, and this may have made it difficult detect these development changes. Finally, the conduct problems subscale of the SDQ measure showed very little internal consistency. It will be important to replicate our results using not only self-report measures of behavioral problems but also with the concurrent use of the parent form in future studies.

With these cautions in mind, our results support continued study of the emotional processing styles associated with CU traits and conduct problems. It adds to the growing body of research suggesting that CU traits are more specifically associated with problems with emotional recognition than conduct problems. Furthermore, our results suggest that the focus of this research needs to be expanded beyond the study of deficits in the emotional recognition accuracy of fearful and sad faces to consider the role of anger and to consider emotional biases (i.e., the tendency to interpret emotional faces as a specific type of emotion). Specifically, our results not only advance those of past work documenting that CU traits are associated with lower accuracy in identifying emotional faces; our results also suggest that these traits are specifically associated lower accuracy in interpreting fearful and angry faces and with interpreting fearful faces as angry. This latter anger bias, if replicated in other studies, could suggest that those with strong CU traits are primed not only to miss cues of fear and distress in others, as suggested by many theories for the development of CU traits [45], but also to interpret these emotions as an indicator of threats to oneself. Such findings could have important implications not only for the causal theory of CU traits but also for what leads to aggressive behavior in children with CU traits and what deficits should be targeted in the treatment for children that strongly display these traits. That is, interventions for children with CU traits that seek to improve emotional recognition skills and motivate the child to use them in social situations need to consider the tendency to misinterpret emotions as potential threats to oneself. 

## Figures and Tables

**Table 1:** Descriptive statistics and zero-order correlations (Pearson’s *r*).

	M (SD)	Skew.	Kurt.	1	2	3	4	5	6	7	8	9	10	11	12	13	14	15	16	17	18	19	20	21	22	23	24	25
1-Sex	-	-	-	-																								
2-Age				0.06	-																							
3-Conduct Problems	0.43 (0.33)	0.85	0.20	−0.01	0.01	-																						
4-CU Traits	0.81 (0.36)	0.62	0.44	0.21 ***	0.12 *	0.38 ***	-																					
5-Emotion Recognition Accuracy (all emotions-frontal)	0.52 (0.19)	−0.54	−0.32	−0.26 ***	0.07	−0.11	−0.17 **	-																				
6-Emotion Recognition Accuracy (all emotions: oriented)	0.48 (0.16)	−0.53	−0.15	−0.29 ***	0.07	−0.12 *	−0.22 ***	0.80 ***	-																			
7-Emotion Recognition Accuracy (all emotions: total)	0.50 (0.17)	−0.67	−0.09	−0.29 ***	0.08	−0.12 *	−0.20 ***	-	-	-																		
8-Emotion Recognition Accuracy (anger: frontal)	0.43 (0.23)	0.17	−0.67	−0.19 ***	0.003	−0.05	−0.15 **	0.66 ***	0.55 ***	-	-																	
9-Emotion Recognition Accuracy (anger: oriented)	0.39 (0.21)	0.09	−0.83	−0.22 ***	0.02	−0.08	−0.16 **	0.57 ***	0.79 ***	-	0.52 ***	-																
10-Emotion Recognition Accuracy (anger: total)	0.43 (0.19)	−0.23	−0.69	−0.24 ***	0.02	−0.09	−0.20 ***	-	-	0.83 ***	-	-	-															
11-Emotion Recognition Accuracy (fear: frontal)	0.45 (0.22)	−0.12	−0.81	−0.27 ***	0.10	−0.02	−0.12 *	0.88 ***	0.70 ***	-	0.52 ***	0.47 ***	-	-														
12-Emotion Recognition Accuracy (fear: oriented)	0.50 (0.23)	−0.23	−0.71	−0.27 ***	0.09	−0.06	−0.18 **	0.71 ***	0.86 ***	-	0.46 ***	055 ***	-	0.66 ***	-													
13-Emotion Recognition Accuracy (fear: total)	0.46 (0.21)	−0.31	−0.83	−0.29 ***	0.11	−0.04	−0.15 **	-	-	0.91 ***	-	-	0.68 ***	-	-	-												
14-Emotion Recognition Bias (anger: frontal)	0.20 (0.15)	0.67	−0.31	−0.07	0.08	0.03	0.11	0.11	0.02	-	0.14 *	0.01	-	0.20 ***	0.03	-	-											
15-Emotion Recognition Bias (anger: oriented)	0.22 (0.15)	0.56	0.02	0.004	−0.06	0.004	−0.04	−0.10	−0.10	-	0.01	−0.29 ***	-	−0.09	−0.01	-	0.23 ***	-										
16-Emotion Recognition Bias (anger: total)	0.20 (0.12)	0.59	−0.01	−0.04	0.01	−0.003	0.04	-	-	−0.001	-	-	−0.20 ***	-	-	0.08	-	-	-									
17-Emotion Recognition Bias (fear: frontal)	0.20 (0.15)	0.38	−0.23	0.07	−0.11	−0.13*	−0.04	−0.19 ***	−0.15 **	-	−0.07	−0.06	-	−0.40 ***	−0.15 **	-	−0.32 ***	−0.01	-	-								
18-Emotion Recognition Bias (fear: oriented)	0.16 (0.11)	0.43	−0.01	0.01	−0.10	−0.04	−0.004	−0.14 *	−0.14 *	-	−0.01	0.02	-	−0.13 **	−0.38 ***	-	0.01	−0.13 *	-	0.12 *	-							
19-Emotion Recognition Bias (fear: total)	0.19 (0.10)	0.19	−0.43	0.06	−0.15 **	−0.11	−0.04	-	-	−0.23 ***	-	-	−0.03	-	-	−0.42 ***	-	-	−0.22 ***	-	-	-						
20-Specific Emotion Recognition Bias (anger to fear: frontal)	0.76 (1.16)	2.16	5.71	0.03	0.02	0.10	0.25 ***	−0.46 ***	−0.42 ***	-	−0.28 ***	−0.34 ***	-	−0.42 ***	−0.37 ***	-	0.44 ***	0.26 ***	-	−0.08	0.10	-	-					
21-Specific Emotion Recognition Bias (anger to fear: oriented)	0.91 (1.18)	2.01	5.48	0.10	−0.09	0.14 *	0.17 **	−0.49 ***	−0.52 ***	-	−0.34 ***	−0.48 ***	-	−0.41 ***	−0.49 ***	-	0.16 **	0.50 ***	-	0.04	−0.03	-	0.50 ***	-				
22-Specific Emotion Recognition Bias (anger to fear: total)	1.67 (2.02)	2.10	5.53	0.08	−0.04	0.14 *	0.24 ***	-	-	−0.57 ***	-	-	−0.58 ***	-	-	−0.53 ***	-	-	0.49 ***	-	-	0.02	-	-	-			
23-Specific Emotion Recognition Bias (fear to anger: frontal)	0.92 (1.16)	1.88	5.52	0.02	−0.04	0.004	0.12 *	−0.40 ***	−0.35 ***	-	−0.46 ***	−0.28 ***	-	−0.39 ***	−0.28 ***	-	−0.06	0.11	-	0.48 ***	0.09	-	0.22 ***	0.22 ***	-	-		
24-Specific Emotion Recognition Bias (fear to anger: oriented)	0.62 (0.98)	2.58	11.93	0.03	−0.07	−0.004	0.11	−0.35 ***	−0.39 ***	-	−0.23 ***	−0.35 ***	-	−0.30 ***	−0.38 ***	-	−0.03	0.05	-	0.14 *	0.54 ***	-	0.29 ***	0.13 *	-	0.36 ***	-	
25-Specific Emotion Recognition Bias (fear to anger: total)	1.54 (1.77)	2.07	7.92	0.03	−0.07	0.001	0.14 *	-	-	−0.48 ***	-	-	−0.46 ***	-	-	−0.44 ***	-	-	0.03	-	-	0.47 ***	-	-	0.30 ***	-	-	-

Notes. * *p* < 0.05, ** *p* < 0.01, *** *p* < 0.001.

**Table 2:** Linear regression analyses (standardized β).

	Sex	Age	Conduct Problems	CU Traits	R^2^	F
Emotion recognition accuracy (all emotions: total)	−0.27 ***	0.11	−0.07	−0.13 *	0.11	(4,292) = 9.872 ***
Emotion recognition accuracy (anger: total)	−0.21 ***	0.05	−0.04	−0.15 * (a)	0.07	(4,292) = 6.580 ***
Emotion recognition accuracy (fear: total)	−0.28 ***	0.14 *	−0.01	−0.11	0.10	(4,292) = 9.135 ***
Emotion recognition bias (anger: total)	−0.05	0.01	−0.03	0.06	(4,292) = 0.324	0.001
Emotion recognition bias (fear: total)	0.06	−0.15 *	−0.11	0.01 (b)	0.02	(4,292) = 2.794 *

Notes. * *p* < 0.05, *** *p* < 0.001. Sex: 0 = girls, 1 = boys. (a) In Step 2 of the multiple regression analysis, there is a significant two-way conduct problems × CU traits interaction term: β = 0.13 *; F(5,292) = 6.381 ***; R^2^ = 0.08, ΔR^2^ = 0.01 *. (b) There is, in Step 4 of the multiple regression analysis, a significant three-way interaction term conduct problems × CU traits × sex: β = 0.19 *; F(8,292) = 2.546 *; R^2^ = 0.04, ΔR^2^ = 0.02 *.

**Table 3:** Negative binomial regression analyses (B).

	Sex	Age	Conduct Problems	CU Traits	Pseudo R^2^	LR χ^2^ (df)
Specific emotion recognition bias (anger to fear: total)	−0.06	−0.07	0.23	0.70 *	0.01	16.642 ** (4)
Specific emotion recognition bias (fear to anger: total)	0.03	−0.11	−0.22	0.55	0.02	6.620 (4)

Notes. * *p* < 0.05, ** *p* < 0.01.

## Data Availability

There are no unpublished data available. The corresponding author can be contacted regarding this matter.

## References

[1] Frick P.J., Ray J.V., Thornton L.C., Kahn R.E. (2014). Can callous-unemotional traits enhance the understanding, diagnosis, and treatment of serious conduct problems in children and adolescents? A comprehensive review. Psychol. Bull..

[2] Frick P.J. (2022). Some critical considerations in applying the construct of psychopathy to research and classification of childhood disruptive behavior disorders. Clin. Psychol. Rev..

[3] American Psychiatric Association (2013). Diagnostic and Statistical Manual of Mental Disorders.

[4] Ciucci E., Baroncelli A., Franchi M., Golmaryami F.N., Frick P.J. (2014). The association between callous-unemotional traits and behavioral and academic adjustment in children: Further validation of the Inventory of Callous-Unemotional Traits. J. Psychopathol. Behav. Assess..

[5] Ciucci E., Baroncelli A., Golmaryami F.N., Frick P.J. (2015). The emotional correlates to callous-unemotional traits in children. J. Child. Fam. Stud..

[6] De Brito S.A., Forth A.E., Baskin-Sommers A.R., Brazil I.A., Kimonis E.R., Pardini D., Frick P.J., Blair R.J.R., Viding E. (2021). Psychopathy. Nat. Rev. Dis. Primers.

[7] Soto C.J., John O.P., Gosling S.D., Potter J. (2011). Age differences in personality traits from 10 to 65: Big Five domains and facets in a large cross-sectional sample. J. Personal. Soc. Psychol..

[8] de Castro B.O., Merk W., Koops W., Veerman J.W., Bosch J.D. (2005). Emotions in social information processing and their relations with reactive and proactive aggression in referred aggressive boys. J. Clin. Child Adolesc. Psychol..

[9] Dodge K.A., Somberg D.R. (1987). Hostile attributional biases among aggressive boys are exacerbated under conditions of threats to the self. Child Dev..

[10] Hartman R., Stage S.A. (2000). The relationship between social information processing and in-school suspension for students with behavioral disorders. Behav. Disord..

[11] Hartmann D., Ueno K., Schwenck C. (2020). Attributional and attentional bias in children with conduct problems and callous-unemotional traits: A case-control study. Child Adolesc. Psychiatry Ment. Health.

[12] Lemerise E.A., Arsenio W.F. (2000). An integrated model of emotion processes and cognition in social information processing. Child Dev..

[13] Marsh A.A., Blair R.J.R. (2008). Deficits in facial affect recognition among antisocial populations: A meta-analysis. Neurosci. Biobehav. Rev..

[14] Bedford R., Carter Leno V., Wright N., Bluett-Duncan M., Smith T.J., Anzures G., Pickles A., Sharp H., Hill J. (2021). Emotion recognition performance in children with callous-unemotional traits is modulated by co-occurring autistic traits. J. Clin. Child Adolesc. Psychol..

[15] Blair R.J.R., Budhani S., Colledge E., Scott S. (2005). Deafness to fear in boys with psychopathic tendencies. J. Child Psychol. Psychiatry.

[16] Blair R.J.R., Coles M. (2000). Expression recognition and behavioural problems in early adolescence. Cogn. Dev..

[17] Blair R.J.R., Colledge E., Murray L., Mitchell D.G. (2001). A selective impairment in the processing of sad and fearful expressions in children with psychopathic tendencies. J. Abnorm. Child Psychol..

[18] Dadds M.R., El Masry Y., Wimalaweera S., Guastella A.J. (2008). Reduced eye gaze explains “fear blindness” in childhood psychopathic traits. J. Am. Acad. Child Adolesc. Psychiatry.

[19] Kimonis E.R., Frick P.J., Fazekas H., Loney B.R. (2006). Psychopathy, aggression, and the processing of emotional stimuli in non-referred girls and boys. Behav. Sci. Law.

[20] Martin-Key N.A., Graf E.W., Adams W.J., Fairchild G. (2018). Facial emotion recognition and eye movement behaviour in conduct disorder. J. Child Psychol. Psychiatry.

[21] Muñoz L.C. (2009). Callous-unemotional traits are related to combined deficits in recognizing afraid faces and body poses. J. Am. Acad. Child Adolesc. Psychiatry.

[22] Powell T., Plate R.C., Miron C.D., Wagner N.J., Waller R. (2023). Callous-unemotional traits and emotion recognition difficulties: Do stimulus characteristics play a role?. Child Psychiatry Hum. Dev..

[23] Woodworth M., Waschbusch D. (2008). Emotional processing in children with conduct problems and callous/unemotional traits. Child Care Health Dev..

[24] Billeci L., Muratori P., Calderoni S., Chericoni N., Levantini V., Milone A., Nocentini A., Papini M., Ruglioni L., Dadds M. (2019). Emotional processing deficits in Italian children with disruptive behavior disorder: The role of callous unemotional traits. Behav. Res. Ther..

[25] Seara-Cardoso A., Viding E., Lickley R.A., Sebastian C.L. (2015). Neural responses to others’ pain vary with psychopathic traits in healthy adult males. Cogn. Affect. Behav. Neurosci..

[26] Carter Leno V., Pickard H., Cybulska L., Smith T., Munafo M., Penton-Voak I., Simonoff E., Pickles A., Bedford R. (2023). Associations between emotion recognition and autistic and callous-unemotional traits: Differential effects of cueing to the eyes. J. Child Psychol. Psychiatry.

[27] Dawel A., O’Kearney R., McKone E., Palermo R. (2012). Not just fear and sadness: Meta-analytic evidence of pervasive emotion recognition deficits for facial and vocal expressions in psychopathy. Neurosci. Biobehav. Rev..

[28] Winters D.E., Sakai J.T. (2022). Emotion Identification for self and other associated with callous-unemotional traits and sex differences in early adolescents. Child Psychiatry Hum. Dev..

[29] Aspan N., Bozsik C., Gadoros J., Nagy P., Inantsy-Pap J., Vida P., Halasz J. (2014). Emotion recognition pattern in adolescent boys with attention-deficit/hyperactivity disorder. Biomed. Res. Int..

[30] Leist T., Dadds M.R. (2009). Adolescents’ ability to read different emotional faces relates to their history of maltreatment and type of psychopathology. Clin. Child Psychol. Psychiatry.

[31] Jones A.P., Laurens K.R., Herba C.M., Barker G.J., Viding E. (2009). Amygdala hypoactivity to fearful faces in boys with conduct problems and callous-unemotional traits. Am. J. Psychiatry.

[32] Schwenck C., Gensthaler A., Romanos M., Freitag C.M., Schneider W., Taurines R. (2014). Emotion recognition in girls with conduct problems. Eur. Child Adolesc. Psychiatry.

[33] Sharp C., Vanwoerden S., Van Baardewijk Y., Tackett J.L., Stegge H. (2015). Callous-unemotional traits are associated with deficits in recognizing complex emotions in preadolescent children. J. Personal. Disord..

[34] Hartmann D., Schwenck C. (2020). Emotion processing in children with conduct problems and callous-unemotional traits: An investigation of speed, accuracy, and attention. Child Psychiatry Hum. Dev..

[35] Berluti K., Ploe M.L., Marsh A.A. (2023). Emotion processing in youths with conduct problems: An fMRI meta-analysis. Transl. Psychiatry.

[36] Essau C.A., Sasagawa S., Frick P.J. (2006). Callous-unemotional traits in a community sample of adolescents. Assessment.

[37] Fine S.E., Trentacosta C.J., Izard C.E., Mostow A.J., Campbell J.L. (2004). Anger perception, caregivers’ use of physical discipline, and aggression in children at risk. Soc. Dev..

[38] Barth J.M., Bastiani A. (1997). A longitudinal study of emotion recognition and preschool children’s social behavior. Merrill-Palmer Q..

[39] Cook E.T., Greenberg M.T., Kusche C.A. (1994). The relations between emotional understanding, intellectual functioning, and disruptive behavior problems in elementary-school-aged children. J. Abnorm. Child Psychol..

[40] Crick N.R., Dodge K.A. (1994). A review and reformulation of social information-processing mechanisms in children’s social adjustment. Psychol. Bull..

[41] Schultz D., Izard C.E., Ackerman B.P. (2000). Children’s anger attribution bias: Relations to family environment and social adjustment. Soc. Dev..

[42] Hodsoll S., Lavie N., Viding E. (2014). Emotional attentional capture in children with conduct problems: The role of callous-unemotional traits. Front. Hum. Neurosci..

[43] Kimonis E.R., Frick P.J., Muñoz L.C., Aucoin K.J. (2008). Callous-unemotional traits and the emotional processing of distress cues in detained boys: Testing the moderating role of aggression, exposure to community violence, and histories of abuse. Dev. Psychopathol..

[44] Waller R., Hyde L.W. (2018). Callous-unemotional behaviors in early childhood: The development of empathy and prosociality gone awry. Curr. Opin. Psychol..

[45] Frick P.J., Kemp E.C. (2021). Conduct disorders and empathy development. Annu. Rev. Clin. Psychol..

[46] Hall J.K., Hutton S.B., Morgan M.J. (2010). Sex differences in scanning faces: Does attention to the eyes explain female superiority in facial expression recognition?. Cogn. Emot..

[47] Senju A., Johnson M.H. (2009). The eye contact effect: Mechanisms and development. Trends Cogn. Sci..

[48] Rigato S., Menon E., Farroni T., Johnson M.H. (2013). The shared signal hypothesis: Effects of emotion-gaze congruency in infant and adult visual preferences. Br. J. Dev. Psychol..

[49] Bayliss A.P., Frischen A., Fenske M.J., Tipper S.P. (2007). Affective evaluations of objects are influenced by observed gaze direction and emotional expression. Cognition.

[50] Shimojo S., Simion C., Shimojo E., Scheier C. (2003). Gaze bias both reflects and influences preference. Nat. Neurosci..

[51] Adams R.B., Kleck R.E. (2003). Perceived gaze direction and the processing of facial displays of emotion. Psychol. Sci..

[52] Adams R.B., Kleck R.E. (2005). Effects of direct and averted gaze on the perception of facially communicated emotion. Emotion.

[53] Akechi H., Senju A., Kikuchi Y., Tojo Y., Osanai H., Hasegawa T. (2009). Does gaze direction modulate facial expression processing in children with autism spectrum disorder?. Child. Dev..

[54] Istituto Nazionale di Statistica Livelli di Istruzione e ritorni Occupazionali—Anno 2021. https://www.istat.it/it/files/2022/10/Livelli-di-istruzione-e-ritorni-occupazionali-anno-2021.pdf.

[55] Kemp E.C., Ray J.V., Frick P.J., Robertson E.L., Fanti K.A., Essau C.A., Baroncelli A., Ciucci E., Bijttebier P. (2023). Inventory of Callous-Unemotional Traits (ICU). Factor structure and measurement invariance in an adolescent multinational sample. J. Clin. Child. Adolesc. Psychol..

[56] Goodman R., Meltzer H., Bailey V. (1998). The Strengths and Difficulties Questionnaire: A pilot study on the validity of the self-report version. Eur. Child Adolesc. Psychiatry.

[57] Ortuno-Sierra J., Chocarro E., Fonseca-Pedrero E., i Riba S.S., Muñiz J. (2015). The assessment of emotional and Behavioural problems: Internal structure of The Strengths and Difficulties Questionnaire. Int. J. Clin. Health Psychol..

[58] van Widenfelt B.M., Goedhart A.W., Treffers P.D., Goodman R. (2003). Dutch version of the Strengths and Difficulties Questionnaire (SDQ). Eur. Child Adolesc. Psychiatry.

[59] Goodman R., Renfrew D., Mullick M. (2000). Predicting type of psychiatric disorder from Strengths and Difficulties Questionnaire (SDQ) scores in child mental health clinics in London and Dhaka. Eur. Child Adolesc. Psychiatry.

[60] Dalrymple K.A., Gomez J., Duchaine B. (2013). The Dartmouth Database of Children’s Faces: Acquisition and validation of a new face stimulus set. PLoS ONE.

[61] Bennetts R.J., Murray E., Boyce T., Bate S. (2017). Prevalence of Face Recognition Deficits in Middle Childhood. Q. J. Exp. Psychol..

[62] Della Longa L., Nosarti C., Farroni T. (2022). Emotion Recognition in Preterm and Full-Term School-Age Children. Int. J. Environ. Res. Public Health.

[63] Holmbeck G.N. (2002). Post-hoc probing of significant moderational and mediational effects in studies of pediatric populations. J. Pediat. Psychol..

[64] McClure E.B. (2000). A meta-analytic review of sex differences in facial expression processing and their development in infants, children, and adolescents. Psychol. Bull..

[65] Blair R.J. (1995). A cognitive developmental approach to morality: Investigating the psychopath. Cognition.

[66] Waller R., Wagner N. (2019). The Sensitivity to Threat and Affiliative Reward (STAR) model and the development of callous-unemotional traits. Neurosci. Biobehav. Rev..

